# Synthesis and Biological Activities on Metal Complexes of 2,5-Diamino-1,3,4-thiadiazole Derived from Semicarbazide Hydrochloride

**DOI:** 10.3390/molecules16075861

**Published:** 2011-07-12

**Authors:** Joshua A. Obaleye, Johnson F. Adediji, Matthew A. Adebayo

**Affiliations:** 1 Department of Chemistry, University of Ilorin, Ilorin, Kwara State, Nigeria; Email: jobaleye@yahoo.com; Tel.: +2348033582048; 2 Department of Chemical Sciences, Ajayi Crowther University, Oyo State, Nigeria

**Keywords:** antimicrobial, toxicological, semicarbazide hydrochloride, ligand

## Abstract

A bioactive ligand, 2,5-diamino-1,3,4-thiadiazole (**L**), derived from semicarbazide hydrochloride, and its metal complexes were prepared and characterized. The complexes were characterized using elemental, infra-red, ultraviolet/visible, magnetic moment, atomic absorption, thin layer chromatography and molar conductance measurements. The IR data revealed that the ligand (**L**) behaved as a tridentate neutral ligand. It coordinated to the metal ion via sulphur and nitrogen of the amines. The molar conductance data reveal that the chelates are non-electrolytes. From the Ultraviolet/Visible spectra and magnetic moment data, the complexes were found to have octahedral geometrical structure. *In vivo* evaluation of the antimicrobial activities of the metal complexes and the ligands showed greater activity against some micro-organisms when compared to the parent compounds. The chelates do not show toxicity against the activities of enzymes from homogenates of liver, kidney and serum in experimental rats.

## 1. Introduction

Aminourea based derivatives exhibit a range of bioactivities, including anti-angiogenic, anti-tumour, anti-malarial, anti-inflammatory and analgesic [1,2,3], anti-tubercular, anti-glaucoma, anti-HIV, cytotoxic and antimicrobial properties [4,5,6].

The synthesis of metal aminourea derivatives had received much attention due to the fact that they are among the first effective chemotherapeutic agents to be employed for the prevention and cure of bacterial infection in humans. The pharmacological activity of these types of molecules is often enhanced by complexation with metal ions [7,8]. The antibacterial activity of aminourea is confined only to micro-organisms which synthesize their own folic acid [9]. The effectiveness of burn treatment seemed to depend not only on the presence of metal ions but also crucially on the nature of the material to which the metal ion is bound [10]. Certain theories had been advanced advocating that a major portion of drug action occurred through complexation [11].

The importance of metal ions in biological systems is well known. One of the most interesting features of metal coordinated systems is the concerted spatial arrangement of the ligands around the metal ion. Among metal ions of biological importance, Cu (II) ion presents a high number of complexes with distortion [12].

In recent years, several aminourea derivatives have been synthesized and their biological activities have been explored [13], but few experimental data about their antimicrobial and toxicological activities have been reported. The present study sought to investigate the physicochemical and biological activities of some novel aminourea derivatives. In continuation of our work on biologically important ligand metal complexes [14,15], this work therefore involves the synthesis, characterization and biological studies of Co(II), Ni(II) and Cu(II) complexes with 2,5-diamino-1,3,4-thiadiazole.

## 2. Results and Discussion

### 2.1. Preparation and Characterization of the Ligand

**Scheme 1:**
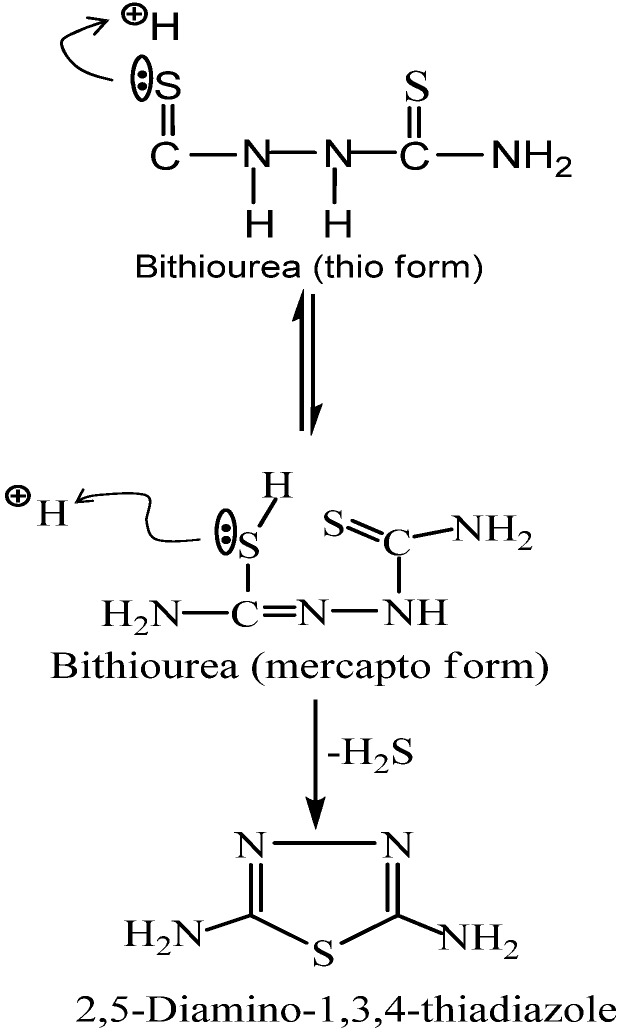
Mechanism for cyclisation of bithiourea.

The cyclisation of bithiourea was performed by 3% hydrogen peroxide (H_2_O_2_). The probable mechanism of this cyclisation is shown in Scheme 1 below. Compound **L** was separated in high yield (96.4%). The structure of the ligand **L** was elucidated based on elemental (Table 1) and spectral data. Its IR spectra (Table 2) showed the absorption bands of NH_2_ and C−S at 3,195 and 1,430 cm^−1^, respectively. 

The aim of this study was to investigate the chelating properties of the 2,5-diamino-1,3,4-thiadiazole ligand towards some biologically important metals like Co(II), Ni(II) and Cu(II) and assign the possible structures of these complexes. The results of the elemental analyses (C, H, N, S and metal content) with the proposed molecular formulae are presented in Table 1. The results obtained are in good agreement with those calculated for the suggested formulae, 1:2 (M:L) solid chelates are isolated and found to have the general formulae [(ML_2_)]X_2_; M=Co(II), Ni(II) and Cu(II) (X=Cl). The solid complexes are prepared and characterized by different tools of analyses like IR, molar conductance, magnetic moment, UV/Visible (Table 3) and atomic absorption spectroscopy to throw more light on the coordination behaviour of this ligand towards some biologically active metals under study.

**Table 1:** Magnetic moment and elemental data of Ligand and their metal complexes.

Compound	Empiricalformula	Formulaweight	μ_eff_ (BM)	Elemental Analysis Calculated (Found)				
				C	H	N	S	Me
**L**	C_2_H_4_N_4_S	116.00	–	20.69 (20.67)	3.45 (3.42)	48.28 (48.22)	13.79 (13.73)	–
**Co(L)_2_Cl_2_**	CoC_4_H_8_S_2_Cl_2_	361.50	4.72	13.28 (13.24)	2.21 (2.20)	30.98 (30.97)	17.22 (17.20)	16.04 (16.00)
**Ni(L)_2_Cl_2_**	NiC_4_H_8_S_2_Cl_2_	361.00	4.26	13.30 (13.29)	2.22 (2.21)	31.02 (31.01)	17.73 (17.70)	16.07 (16.02)
**Cu(L)_2_Cl_2_**	CuC_4_H_8_S_2_Cl_2_	367.00	1.90	13.08 (13.04)	2.18 (2.15)	30.52 (30.50)	17.44 (17.40)	17.44 (17.40)

**Table 2:** IR spectral assignment of L and its metal complexes.

Ligand/complexes	υ(NH_2_)	υ(C–S) cm^−1^	Δ(NH_2_) cm^−1^
**L**	3195.31,b	1430, str.	1536.55,str.
**Co(L)_2_Cl_2_**	3414.06,b	1429.98,s	1536.92,s
**Ni(L)_2_Cl_2_**	3414.48,b	1429.82,s	1537.62,s
**Cu(DT)_2_Cl_2_**	3424.82, b	1407.37,s	

**Table 3:** Ultraviolet/visible spectral assignment of L and its metal complexes [wavelength, nm (cm^−1^)].

Compound	Band 1	Band 2	Band 3	Band 4
**L**	205(48780)	238(42017)	–	–
**Co(L)_2_Cl_2_**	232(43103)	271(36900)	526(19011)	529(18904)
**Ni(L)_2_Cl_2_**	229(43668)	256(39063)	343(29155)	817(12240)
**Cu(L)_2_Cl_2_**	229(43668)	277(36101)	361(27700)	364(27473)

The metal chloride salts react with the ligand **L** (**L** = 2,5-diamino-1,3,5-thiadiazole) according to the following proposed general equation: [M(II)L_2_]Cl_2_, where M = Cu^2+^, Co^2+^ and Ni^2+^ metal salt. The complexes synthesized were found to be non-hygroscopic solids with light green, peach and green colours, respectively (Table 4). The complexes are well soluble in DMSO and DMF and hot distilled water. They have sharp melting points, and no decomposition was observed. The average percentage yield was very high. The retention factor (R_f_) values was calculated from the developed single spot for the complexes indicating the purity of the compound [16]. The retention factor of the metal complexes was found to be higher than the ligand. The analytical data of the metal complexes showed 1:2 stoichiometry.

**Table 4:** Physical properties of L and its metal complexes.

Compounds	Melting point (°C)	Colour	% Yield	Conductivity (Ω^−1^ cm^−1^ dm^−3^)
**L**	208	White	96.4	
**Co(L)_2_Cl_2_**	190	Peach	75.2	1.7 × 10^−6^
**Ni(L)_2_Cl_2_**	200	Green	61.3	1.3 × 10^−6^
**Cu(L)_2_Cl_2_**	140	Light green	87.3	2.4 × 10^−6^

### 2.2. Infrared Spectra and Mode of Bonding

The IR spectra of the free ligand and their metal complexes were recorded in the range of 4,000–400 cm^−1^ and are listed in Table 2. The assignments have been carried out based on literature values obtained for structurally similar compounds [21]. The important IR frequencies of the ligand **L** and the metal complexes (in KBr) with their tentative assignments are given. Both the free ligand and the metal complexes are characterized by υ(N−H), δ(NH_2_), υ(C−S) bands [22]. The absorption patterns look quite similar to that of the free ligand which is in agreement with coordination through nitrogen atom. The band around 3,400–3,100 cm^−1^ is assigned to υ(NH) and is supported by the presence of δ(NH_2_) deformation bands around 1,600–1,500 cm^−1^. A blue shift was observed in the υ(C−S) frequency of the complexes, in comparison to the free ligand, which indicates coordination through the sulphur atom. Bands between 800–900 cm^−1^ which were absent in the free ligand, are assigned to M-L and are due to the metal-ligand coordination. Using the IR spectra, it is concluded that the ligand **L** behaves as a neutral tridentate ligand. It coordinated to the metal ions via the nitrogen of the amines and sulphur atom.

### 2.3. Molar Conductance Data

The molar conductance of the solid complexes (λ_m_, Ω^−1^ cm^2^ mol^−1^) was calculated. The DMF solubility of the above complexes made calculations of the molar conductivity (λ_m_) of 10^−3^ mol dm^−3^ solution at 25 °C possible. The data in Table 4 showed that the molar conductance values of the Co(II), Ni(II), and Cu(II) complexes were relatively low, indicating the non-electrolytic nature of these complexes.

### 2.4. UV/Visible Spectra and Magnetic Moments

For the Co(II) complex with ligand **L** the UV/visible spectra showed two bands in the visible region at 19,011 cm^−1^ and 18,904 cm^−1^, which were assigned to ^4^T_1g_ (F) → ^4^T_2g_ (F) and ^4^T_1g_ (F) → ^4^E_2g_ (F) transitions, respectively, which assume an octahedral geometry for Co(II) complex [25,26,27]. The two bands at 43,103 and 36,900 cm^−1^ refer to LMCT [24]. The Co(II) complex had μ_eff_ of 4.69–4.92 BM which assumed a high spin octahedral geometry [23], that may arise from spin-spin coupling and/or crystal distortion.

The electronic spectra of the Ni(II) complex of the 2,5-diamino-1,3,4-thiadiazole ligand displays one band in the visible region at 12,240 cm^−1^ which is assigned to ^3^A_2g_ → ^3^T_2g_. The bands at 43,668 cm^−1^, 39,063 cm^−1^ and 29,155 cm^−1^ refers to LMCT [24]. This indicates the octahedral geometry of the Ni(II) complex [23]. The Ni(II) complex has μ_eff_ of 3.53–4.26 BM, which suggest an octahedral geometry.

The UV/visible of the Cu(II) complex consists of a broad and low intensity shoulder band at 27,473–43,668 cm^−1^ that forms part of the charge transfer band. The ^2^E_g_ and ^2^T_2g_ states of the octahedral Cu(II) ion (d^9^) split under the influence of the tetragonal distortion into three transitions and remain unresolved in the spectra. It is concluded that all three transitions lie within the single broad envelope centred at the same range previously mentioned. This assignment is in agreement with the general observation that Cu(II) d-d transitions are normally close in energy. The Cu(II) complex has μ_eff_ of 1.83–1.96 BM, assuming a distorted octahedral structure [24].

### 2.5. Structural Interpretation

**Figure 1:**
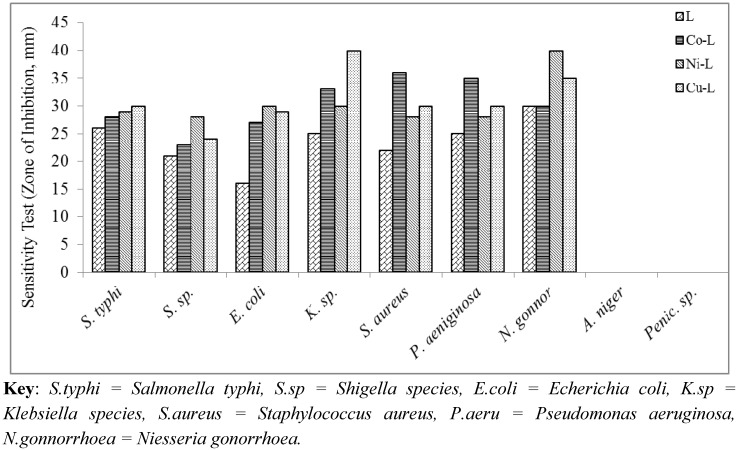
Sensitivity test of the ligand and metal complexes against some micro-organisms.

**Figure 2:**
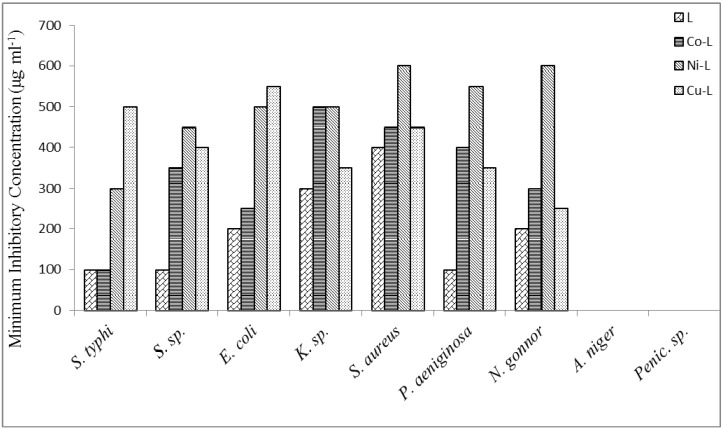
Minimum inhibition concentration of the ligand and metal complexes against some micro-organisms.

**Figure 3:**
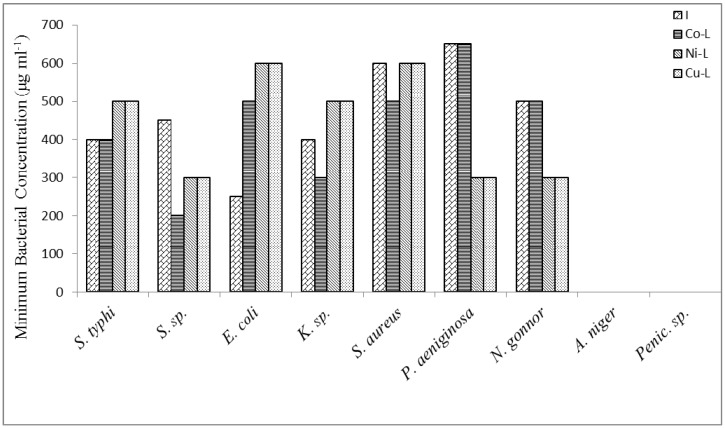
Minimum bactericidal concentration of the ligand and metal complexes against some micro-organisms.

Consequently, the structures proposed are based on octahedral geometric structures. The 2,5-diamino-1,3,4-thiadiazole coordinate via nitrogen of the amines and sulphur atom forming three binding chelating sites. Figure 1, Figure 2 and Figure 3 reported the antimicrobial activity results determined from the *in-vitro* studies of the ligand and its metal complexes. Generally, the ligand and metal complexes showed antimicrobial effect against the tested organisms, except against moulds of penicillin and *Aspergillius* as presented in the figures above. *Niesseria gonorrhoea* was the most sensitive organism to the 2,5-diamino-1,3,4-thiadizole and its metal complexes. Metal complexes showed comparable or greater activity against some of the micro-organisms in comparison to the parent compounds.

The MIC of the samples against the various isolates ranged from 15 μg/mL to 700 μg/mL. These concentrations in comparison to the reported MIC_90_ of the ligand elsewhere are very high. This could be due to the different conditions under which the studies were carried out. These are reflections of the fact of possible interference from the media broth and some other materials and chemicals used during the test, which are not absolutely compatible with conditions present in the cells [27].

For a particular antimicrobial, the organism involved is an important factor; *Salmonella typhi, Shigella species, Pseudomonas aeruginosa* are more sensitive to the metal complexes than *Klebsiella species, Escherichia coli* and *Staphylococcus aureus*. Reports have shown that CuCl_2_2H_2_O, CoCl_2_6H_2_O and NiCl_2_6H_2_O have no inhibitory activity on bacteria and fungi species [21].

**Figure 4:**
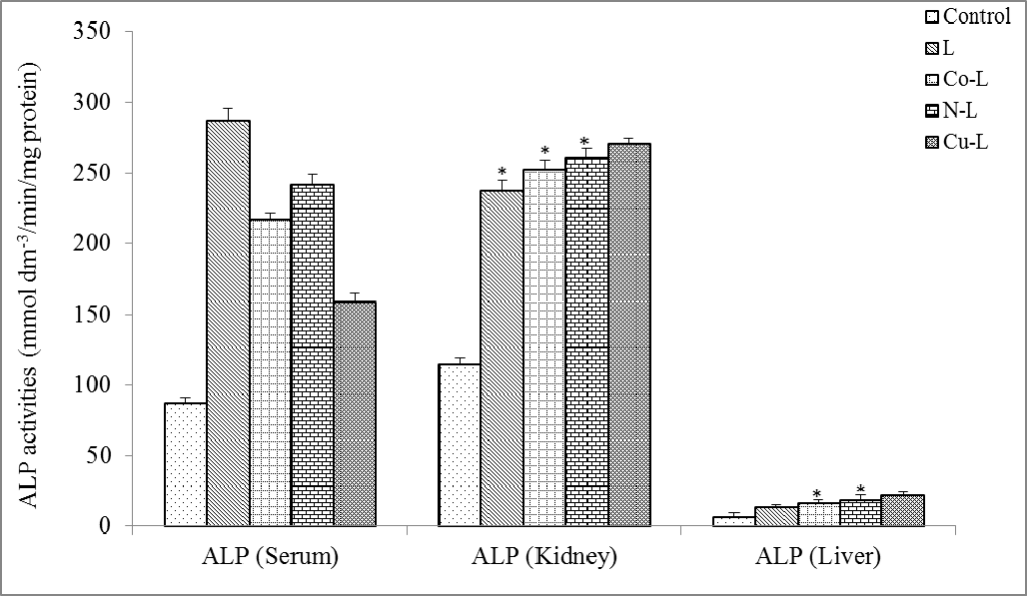
Effect of administration of ligand and the metal complex on the activities of alkaline phosphatase of rat serum, kidney and liver. * Significantly different from the control (p < 0.05).

**Figure 5:**
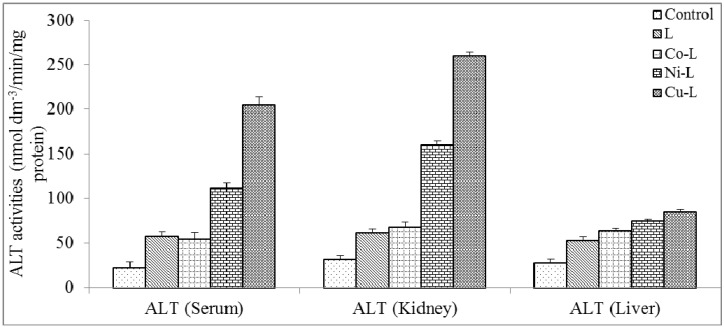
Effect of administration of ligand and the metal complex on the activities of alanine amino transferase (ALT) of rat serum, kidney and liver. * Significantly different from the control (p < 0.05).

**Figure 6:**
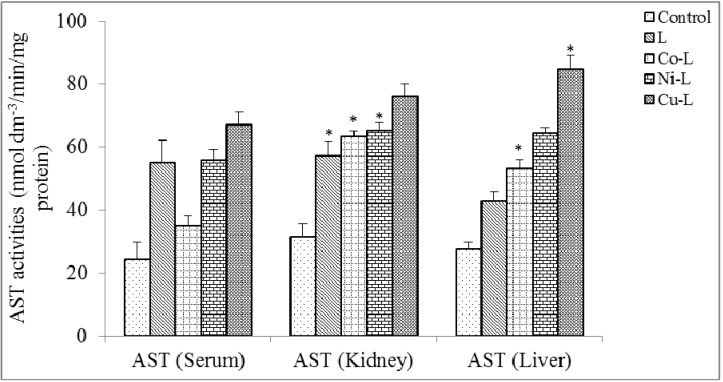
Effect of administration of ligand and the metal complex on the activities of aspartate amino transferase (AST) of rat serum, kidney and liver. * Significantly different from the control (p < 0.05).

Figure 4, Figure 5 and Figure 6 show the results of ALP, ALT and AST activities in the serum, kidney and liver. There was no significant reduction (p < 0.05) in serum ALP activities of 2,5-diamino-1,3,4-thiadiazole and its metal complex compared with control, this suggests that the integrity of the plasma membrane of the cells in the various tissues might have not been adversely affected. This is because ALP is a membrane-bond enzyme often used to assess the integrity of the plasma membrane and endoplasmic reticulum [29]. The observed significant increase in the ALP activities in the liver and kidney of the rat administered with metal complexes suggests an enhancement of the activities of the existing enzymes by the drugs and their metabolites. The increase may be as a result of stress imposed on the tissue by the drug, which may lead to loss of the enzyme molecule through leakage into the extra-cellular fluid, which has been significantly noticed in the serum. In a bid to offset this stress, the tissue may increase the *de novo* synthesis of the enzyme, thus accounting for the increase in activities in these tissues [30]. However the metal complex of Cu(II) caused a significant increase in serum ALT activity compared with control. There was also a significant increase in liver and kidney ALT and AST activities compared with control. Elevation of serum ALT and AST activity is a pointer to leakage from a damaged tissue. Increase in serum ALT and AST has been reported in conditions involving necrosis of hepatocytes [30], myocardial cells, erythrocyte and skeletal muscle cells [31]. Overall, the integrity of the cell membranes of the various tissues (especially kidney and liver) was not adversely affected by the metal complexes. It is established from combined results of the chemical and physical analysis and from previous reports that the ligand (2,5-diamino-1,3,4-thiadiazole) employed in this work coordinated with Co(II), Ni(II) and Cu(II). The metal complexes possess better physical properties than the parent compound. The toxicological studies revealed that the metal complexes are not toxic at the dosage level administered. Based on various activities observed, metal complexes of 2,5-diamino-1,3,4-thiadiazole would be a better therapeutic drug for antibacterial treatment.

## 3. Experimental

### 3.1. General

All chemicals used in the preparation of the complexes and in solutions studies were of the highest purity available. Semicarbazide hydrochloride, potassium thiocyanate and 3% hydrogen peroxide were supplied by Sigma. Co(II), Ni(II) chloride hexahydrate and Cu(II) chloride dihydrate (BDH) were used as received. The organic solvents used such as absolute ethanol and methanol were also obtained from BDH. Elemental analyses (C, H, N, S and Met) were performed at the Pontificia Universidade Catolica, Rio de Janeiro, Brazil. The analyses were repeated twice. The IR spectra were recorded from 4,000–400 cm^−1^ using a Perkin-Elmer SP3-30 FT-IR spectrometer. The spectra were recorded as KBr disks. The molar magnetic susceptibilities of the powdered samples were measured using a Faraday Balance Model 7650 with Hg[Co(SCN)_4_] as calibrant. The ultraviolet/visible analysis was carried out on a Genesys10S V1.200 spectrophotometer. The molar conductance measurements of the complexes were carried out in DMF using a Genway 4200 conductivity meter. Metal contents of the complexes were determined using an Alpha4 Atomic Absorption Spectrophotometer equipped with a PM8251 simple-pen recorder. Thin layer chromatography was carried out using TLC plates coated with silica gel. ALP, ALT, and AST assay kits were obtained from Randox Laboratories Limited (Antrim, United Kingdom). Clinical cultures of the micro-organism used were obtained from the University Teaching Hospital and the Department of Microbiology, University of Ilorin, Ilorin, Nigeria. Albino rats (Wistar strain) were obtained from the Department of Biochemistry, University of Ilorin, Ilorin, Nigeria.

### 3.2. Antimicrobial Screening

The stimulatory or inhibitory activity of the ligand and the metal complexes synthesized were determined according to the procedure previously reported with slight modifications [14,15,16]. The bacterial species used for this test include clinical cultures of *Escherichia coli, Staphylococcus aureus, Klebsiella species, Niesseria gonorrhoea, Salmonella typhi, Shigella species, Penicillium species, Pseudomonas aeruginosa and Aspergillus species*. The antibacterial activities of the compounds were determined using sensitivity test, minimum inhibitory concentration and minimum bacterial concentration. 

### 3.3. Sensitivity Tests: Using Mueller Hinton Agar

Plastic disposable sterile Petri dishes were used. Mueller Hinton agar (20 mL) was poured in and allowed to set. The plates were labelled, and then swabbed with the respective standardized test organism (0.1 mL). Holes (8 mm diameter) were made using a cork borer. The holes were then filled with the test samples (20 μg/mL) and the control (solvent) and left to stand for 1 hr for proper diffusion of the agent into the agar. The plates were kept in an incubator at 37 °C and the zones of inhibition measured after 24 hrs. A plate containing only the agar was also kept in the incubator to determine whether contaminants were present.

### 3.4. Sensitivity Disk Test

Media plates of sensitivity test agar (STA) were prepared and inoculated from overnight slant cultures of the test organisms and spread as uniformly as possible throughout the entire media. The antimicrobial sample solution (10 μg/mL) impregnated disks were then placed on the inoculum media. These were incubated at 35 °C for 24 hrs. Degrees of sensitivity were determined by measuring visible areas of inhibition of growth using the zone reader.

### 3.5. Antifungal Activity Test

The plates were filled with the SDA agar (two-thirds) and the fungi specie inoculated into it and the sample solutions added as in the sensitivity test above.

### 3.6. Determination of the Minimum Inhibitory Concentration (Bactericidal)

To each of a series of sterile stoppered test tubes, a standard volume of medium that supports the growth of the test organism was added; this was followed by the addition of 0.1 mL to 7.0 mL at an interval value of 0.05 mL of each of the antimicrobial metal complexes and ligand solutions representing 10, 15, 20, 25, 30 ,35, 40, 45, 50, 80, 100, 200, 300, 400, 500, 600, 700 μg/mL, respectively, in a final mixture of 10 mL. Standard volume of the inoculum (0.2 mL) of each of the test, one in which the test sample (antimicrobial) was omitted and the other in which the test organism was omitted. All the tubes were incubated at 35 °C for 24 hrs. All tubes that show turbidity (evidence of growth) were removed while those showing no turbidity were subcultured into nutrient broth by transferring a loopful of the culture which have been properly shaken into 10 mL of the broth and incubated for 8 hrs at 35 °C. This broth culture was further subcultured on to nutrient agar media by a single stroke streaking and incubated at 35 °C for 24 hrs. The plates were observed for growth after the period of incubation. The minimum concentration plates showing no growth after this period represents the minimum bacterial concentration (MBC).

### 3.7. Treatment of Animals

Male albino rats (Wistar strain), weighing between 160–180 g were obtained commercially from Department of Biochemistry, University of Ilorin, Ilorin, Nigeria. They were kept in wire meshed cages and fed with commercial rat chow (Bendel Feeds Nigeria Ltd) and supply water *ad libitum*. Thirty six rats were divided into three groups of six rats per group. The first group was used as control and received distilled water. The second group of rats was treated with free ligand (2,5-diamino-1,3,4-thiadiazole) while the third group were subdivided into three groups treated with metal complexes [Cu(L)_2_Cl_2_], [Co(L)_2_Cl_2_] and [Ni(L)_2_Cl_2_]. The distilled water, ligand and solution of metal complex were administered orally to the rats of various groups two times daily for seven days at the dose of 0.60 mg/Kg body weight. The animals were sacrificed 24 hrs after the last treatment.

### 3.8. Preparation of Serum and Tissue Homogenates

The method described by Yakubu *et al*. [17] was used to prepare the serum. The rats were sacrificed by cervical dislocation. Blood samples were collected by cardiac punctures into clean, dry centrifuge tube after which they were left for 10 min at room temperature. The tubes were then centrifuged for 10 min at 3,000 × g in an MSC (Essex, UK) bench centrifuge. The clear supernatant (serum) was aspirated using a Pasteur pipette into clean, dry sample bottles and then frozen overnight before use.

The liver and kidney excised from rat, blotted of blood stains was rinsed in 1.15% KCl and homogenized in four volumes of ice-cold 0.01 mol dm^−3^ potassium phosphate buffer (pH 7.4). The homogenates were centrifuged at 12,500 × g for 15 min at 4 °C and the supernatants, termed the post-mitochondrial fractions (PMF) were aliquoted and used for enzyme assays.

### 3.9. Determination of Serum and Tissue ALP, AST and ALT Activities

Serum and tissue’s ALP, AST and ALT activities were determined using Randox diagnostic kits. Determination of AST and ALT activities were based on the principle described by Relitman and Frankel [18]. ALP activity determination was based on the method of Wright *et al*. [19]. The yellow colour p-nitrophenol formed was monitored at 405 nm. Protein determination of serum and all fractions was estimated by the method of Lowry *et al.* [20] as modified by Yakubu *et al.* [17] using bovine serum albumin as standard.

### 3.10. Statistical Analysis

The data were analyzed using one way ANOVA followed by Duncan multivariable post-hoc test for comparison between control and treated rats in all groups. Values of *p* less than 0.05 were considered statistically significant. 

### 3.11. Preparation of the 2,5-diamino-1,3,4-thiadiazole ligand *(**L**)*

#### 3.11.1. Procedure

Bithiourea (30 g, 0.2 mol) was introduced into a 250 mL round bottomed flask and 3% H_2_O_2_ (40 mL) was added. The mixture was refluxed at 50–60 °C for 1 hr with continuous stirring. The product was then filtered under vacuum and dried at 100 °C in the oven and the percentage crude yield was determined. It was thereafter recrystallised from boiling water. 

#### 3.11.2. Synthesis of the metal complexes

The complex was prepared based on previous reported procedures with slight modifications [15]. An aqueous or ethanolic solution of the metal salt (CuCl_2_2H_2_O, CoCl_2_6H_2_O and NiCl_2_6H_2_O) was mixed with an aqueous ethanolic solution of 2,5-diamino-1,3,4-thiadiazole (which was dissolved in minimum amount of the solvent; 0.01 mol each). The reaction mixture was heated in a 250 mL round bottomed flask for 15 min on a water bath and there was change of colouration, indicating the appearance of the precipitates of the complexes. The reaction mixture was reduced to about one third when the metal complex separated out on cooling. The complex formed was recovered from the solution by filtration. It was washed and recrystallised from ethanol and then dried in vacuum over CaCl_2_.

## 4. Conclusions

It is established from the combined results of the chemical and physical analysis and from previous reports that the ligand 2,5-diamino-1,3,4-thiadiazole (**L**) employed in this work coordinated with Cu, Co and Ni. The metal complexes possess better physical properties than the parent compound. Based on antimicrobial activities reported elsewhere and toxicological data from our experiments, metal complexes of 2,5-diamino-1,3,4-thiadiazole would be better therapeutic drugs for antibacterial treatment.
